# Fully Autonomous Active Self-Powered Point-of-Care Devices: The Challenges and Opportunities

**DOI:** 10.3390/s23239453

**Published:** 2023-11-28

**Authors:** Laura Crivillé-Tena, Jordi Colomer-Farrarons, Pere Ll. Miribel-Català

**Affiliations:** 1 Universitat de Barcelona, 08007 Barcelona, Spain; lcrivite7@alumnes.ub.edu; 2 Discrete-to-Integrated Systems Laboratory (D2In), Electronics and Biomedical Engineering Department, Universitat de Barcelona (UB), Marti i Franques, 1-11, 08028 Barcelona, Spain; peremiribelcatala@ub.edu

**Keywords:** point-of-care, self-powered systems, biosensors, continuous monitoring, biofuel cells

## Abstract

Quick and effective point-of-care (POC) devices have the chance to revolutionize healthcare in developed and developing countries since they can operate anywhere the patient is, with the possibility of obtaining and sending the results to the doctor without delay. In recent years, significant efforts have focused on developing new POC systems that can screen for biomarkers continuously and non-invasively in body fluids to prevent, diagnose, and manage diseases. However, one of the critical challenges left to address is how to power them effectively and sufficiently. In developing countries and rural and remote areas, where there are usually no well-established electricity grids or nearby medical facilities, and using batteries is unreliable or not cost-effective, alternative power sources are the most challenging issue for stand-alone and self-sustained POC devices. Here, we provide an overview of the techniques for used self-powering POC devices, where the sample is used to detect and simultaneously generate energy to power the system. Likewise, this paper introduced the state-of-the-art with a review of different research projects, patents, and commercial products for self-powered POCs from the mid-2010s until present day.

## 1. Introduction

Accurate and early stage disease diagnosis is critical for providing the most effective and life-saving treatments while decreasing the turnaround time (TAT). In developed countries, access to high-tech central laboratories with standard protocols and procedures improves the efficiency and accuracy of diagnostic tests due to sophisticated analyzers and imaging techniques.

However, the availability of these tests is limited, as they require complex and time-consuming processes and trained personnel. Moreover, long waiting times at hospitals and slow TATs for obtaining test results lead to higher anxiety and fear from the patient before a final diagnosis is made [1], worsening the patient experience while slowing down the potential treatment. 

Furthermore, these sophisticated diagnostic platforms are mostly not applicable in developing countries since they generally lack all the necessary requirements, such as laboratory infrastructure and equipment, expert personnel, and access to a reliable electrical supply and clean water. So, currently, many people in developing countries die from diseases that can be controlled or cured in developed countries [1].

From that perspective, point-of-care (POC) testing is valuable for providing immediate healthcare information and in vitro diagnostics. Likewise, it applies in various settings, including accident sites, first-aid posts, doctors’ offices, and emergency vehicles such as ambulances. Additionally, patients can use POC testing to manage and test their health conditions at home, making healthcare more accessible and affordable in developed and developing countries. This technology is critical in less advanced regions, where accurate diagnosis is crucial but often difficult to obtain.

Optimal POC devices should be portable, as small as possible, able to be used by inexperienced patients or personnel, and able to give specific, sensitive, and accurate results, achieving reliable outcomes in a short time and with no sample pretreatment. However, POC tests now face one significant challenge: powering. Ideally, these devices should be self-powered: with the ability to generate their energy, a field of study into which much effort is being put, since, in many cases, access to a suitable traditional power source (electrical grid, batteries) is not straightforward, and the autonomy of these devices could sometimes be more optimal.

This issue is particularly challenging for wearable POCs since they also demand anatomically compliant, stretchable, and flexible power sources. Since powering POC devices with standard batteries becomes an issue in many situations, there is the need for stand-alone and self-powered POC devices, because the electrical supply is often sporadic and (or) not widely accessible [2]. In that sense, POC testing devices are generally divided into three categories according to their power techniques and dependency: (i) power-free, (ii) battery-powered handheld, based mainly on low-power electronics or energy harvesting techniques, and (iii) smartphone-based devices [1].

As the name says, power-free devices (i) operate utterly autonomously, without dependence on any power source or batteries. Chang et al. (2022) [3] developed the first power-free microfluidic chip with an auto-flowing function based on the balance between the gravitational potential and the capillary force to control fluid loading. They integrated it with fiber optic particle plasmon resonance for the POC rapid testing of SARS-CoV-2.

However, for many POC applications, it is still necessary to use external power sources to obtain the full benefits. This is the case with battery-powered handheld (ii) devices, in which the energy needed to power the device is stored in a battery that is charged via an external power source. Grant et al. (2021) [4] created a sample preparation device for handheld POC testing. Operated via a lithium-ion battery, it included a removable chip that stored, mixed, and separated the liquids and reagents according to the assay performed, an electronic control system, and an impedance-based position feedback system for appropriate droplet movement.

Finally, a recently appearing approach is a smartphone-based device, for which smartphones’ current functionalities, sensors, and features allow them to be used as POC devices. Garcia-Bernalt et al. (2022) [5] reported the SMART-LAMP approach in this area. This handheld portable device allows for the monitoring of real-time isothermal amplification reactions, employing colorimetric measurements controlled by a specific app on a smartphone via Bluetooth. 

This work studies the advances in self-powered POC devices based on power-free (i) architectures made in the last decade. 

## 2. Market Opportunities

After introducing the different types of devices and the importance of achieving self-powered POCs, it is time to check the market and how promising this sector is considering numbers, significant investments, profits, and the expectation of future growth provisions. 

According to the yearly market research report by Vision Research Reports [6] and Strategic Market Research [7], the global POC diagnostics market witnessed a compound annual growth rate (CAGR) of 11.78%, valued at USD 37.1 billion in 2021. The growing international popularity of rapid testing devices will push future revenue creation from POC diagnostics to reach USD 68–USD 70 billion by 2030 (Figure 1). Likewise, it is worth noting the division of these devices into two broad segments: (1) infectious POC testing, accounting for 60% of the market, and the (2) non-infectious POC testing, taking a market share of 40%. A special mention is due to the glucose monitoring segment, which holds a significant position in the overall POC market, with a revenue of USD 8624.1 million. In addition, the recent COVID-19 pandemic increased the demand for POC testing kits. 

In 2021, the OTC (over-the-counter) testing sector dominated the market, with a revenue share of prescriptions of 58.4%, and is expected to continue growing in the coming years. These tests are affordable, easy to use, and can be performed at home without requiring skilled professionals. As a result, they are trendy and have captured a significant portion of the market.

Furthermore, by 2030, the hospital industry is expected to grow significantly, to an estimated USD 25.3 billion, due to various factors. These include an increase in the prevalence of chronic diseases, higher hospitalization rates, significant investments in healthcare infrastructure from both the private and public sectors, and an increase in the use of advanced medical equipment. Likewise, during that forecast period, the urgent care and clinic sector is expected to experience rapid growth.

In terms of its impact on different regions of the world, the POC market in 2021 was led by North America, generating a revenue of USD 14.48 billion. By 2030, it is expected to reach USD 32.5 billion. Additionally, Europe expects to hold the second highest position in the world market, with the UK, France, and Germany being the three most dominant countries in the continent’s POC market.

## 3. Current Research and Commercial Products

This section reviews the top products available in terms of self-powered POCs which are energized by their sample. It also highlights this market’s immense opportunities and analyzes the current state-of-the-art technology. Most approaches use biofluids to power the devices and measure or monitor parameters and biomarkers from biofluids, particularly sweat. Although using biofluids to power a system is not new, harvesting energy from the wearer’s body has become more common. Original proposals for devices like cochlear implants and pacemakers intend to use glucose biofuel cells (BFCs) to power them. BFCs are a specific type of fuel cell that convert chemical energy into electrical energy via an electrochemical process using natural proteins or microorganisms as fuel (Details on the technologies’ working principles are in Section 4).

However, the enzymes used in the BFC can degrade, and the electrode would then stop functioning within a few days. The only way to restore the operation of the BFC was to remove the implant, which was highly surgically impractical [8]. Fortunately, many advances have been made in the field in recent years. This review section discusses some of the significant achievements presented so far.

With the increasing number of people diagnosed with diabetes, Valdés-Ramírez et al. (2014) [9] have developed a glucose O_2_ microneedle-based self-powered BFC sensor. This sensor harvests biochemical energy from the wearer’s transdermal fluid and provides power signals proportional to the concentration of the fuel. Typically, minimally invasive glucose biosensors for diabetes management rely on the current response of controlled-potential amperometric devices. However, using self-powered biosensors eliminates the need for a power source and provides power density signals proportional to the level of the fuel/substrate. The BFC (bio)anode and (bio)cathode are integrated into a hollow microneedle array, which allows for continuous non-invasive glucose monitoring without an external potentiostat control.

Similarly, Jeerapan et al. (2016) [10] created self-powered sensors that are highly stretchable, textile-based BFCs. These sensors extract power from perspiration to sense and monitor sweat metabolites. The use of screen-printing technology in their fabrication allows simple ink patterning over highly stretchable surfaces such as fabrics. This technology also allows for the engineering of extra functionalities into the inks, providing high stretchability while retaining high electrical conductivity and good electrochemical performance. By integrating textile-based BFCs, the sensors can obtain biochemical energy from the wearer’s perspiration, sense the biomarker level, and simultaneously display it to the readout without requiring external energy sources. These self-powered sensors hold great promise for enhancing the functionality of clothing and promoting various wearable applications. They enable comfortable and discreet health monitoring. In this case, the authors have developed bioelectronic socks containing nanocomposites with the desired functionalities. These socks can measure glucose and lactate concentrations.

Alternatively, we provide two examples below where the sample itself is not used to generate energy. Different systems are used: a solar cell and generation via NFC wireless link. Zhao et al. (2019) [11] developed a self-powered smartwatch that uses solar energy instead of BFCs. The smartwatch is designed for the continuous and non-invasive monitoring of glucose levels. It comprises flexible photovoltaic cells that harvest and convert solar energy, flexible Zn-MnO_2_ rechargeable batteries for intermediate energy storage, an electrochemical sensor for monitoring the glucose levels in sweat, a printed circuit board (PCB) for controlling the module, and an electronic ink display for real-time monitoring (Figure 2). This integrated system stores the energy the photovoltaic cells produce in the batteries, which powers the entire system, including real-time signal processing and data display, without external power sources.

Bandodkar et al. (2019) [12] developed a battery-free skin-interfaced point-of-care (POC) system powered by sweat to analyze electrochemical, colorimetric, and volumetric parameters simultaneously. The system operates like a biofuel cell, with the desired analytes spontaneously creating electrical signals proportional to their concentration, eliminating the need for a potentiostat. Thus, the simplified electronic complexity enables miniaturization and low-cost modules to include near-field communications (NFC) technology. The system comprises a thin, reusable NFC electronic module and a disposable microfluidic network containing lactate and glucose sensors based on biofuel cell technology. The biofuel cell design involves a voltage amplifier and miniature passive components, which simplifies the electronics and allows for the miniaturization of the system for low-cost sensing and communication functions. The integrated NFC chip prepares the signal for digitalization in the circuit. In contrast to other systems, the NFC electronics operate in a low-power mode with a minimal component count and a small footprint. The schematic in Figure 2 shows how the amplification is based on a simple voltage follower design with a high-frequency filter to eliminate the fluctuations introduced by the electric field of the primary NFC antenna. This NFC electronic subsystem magnetically couples to electrochemical sensors embedded in the disposable microfluidic substrate. The electrochemical sensor is located on a disposable microfluidic substrate that contains all the necessary elements for operation, including sensors, valves, channels, reservoirs, a microchannel with a volume of around 58 μL, and an inlet interface capable of absorbing sweat in the range of 12 to 120 μL/hour per cm^2^ for up to 6 h.

Back to POC systems fully energized by the analyzed sample, Yu et al. (2020) [13] developed a unique type of electronic skin called perspiration-powered electronic skin (PPES). It harvests the energy from human sweat through lactate BFCs, which allows for the continuous monitoring of metabolic biomarkers. The data are wirelessly sent to a user interface through low-energy Bluetooth. The PPES substrate is an ultra-soft polymeric substrate designed to comply with the skin’s modulus of elasticity for accurate biosensing. It has achieved sustainable high-power density, which enables continuous self-powered health monitoring.

In the same line, Ortega et al. (2019) [14] developed a self-powered skin patch that measures sweat conductivity to diagnose cystic fibrosis (CF), a disease that causes mutations on a membrane protein called the cystic fibrosis conductance transmembrane regulator. This protein controls the excretion of chloride in sweat, and, as a result, the sweat of patients with CF contains a higher chloride concentration than that of healthy individuals. The most reliable test for CF diagnosis is the sweat test, which can be performed on newborns as early as 48 h after birth [15].

The patch is designed to measure fluid, mainly sweat, using a paper battery. It is made from glass fiber paper with a high water absorption rate and porosity, allowing for a maximum volume of 15 µL. The fluid in the patch acts as an electrolyte, and the battery output depends on the conductivity of the liquid sample poured into its paper core. This system combines the sensor and the battery into one element, in which the generated power is directly related to the conductivity of the analyzed sample. The patch uses minimal, discrete, and completely printable electronic components to achieve an autonomous screening patch [14]. Sweanty, a recent startup [16], has created two patch products based on this technology: one for CF screening and the other for hydration assessment. 

Along the same line, Jeerapan et al. (2022) [17] fabricated the first example of self-powered mask-based bioelectronics that can measure a biological glucose signal with continuous energy using a small sample volume of less than 100 μL. The mask-based electrodes use a screen-printing technique, a cost-effective method for large-scale production. The (bio)anode produces electrons by reacting the glucose in sweat with the glucose oxidase present on the anode surface. These electrons help drive the self-sustainable energy system and travel toward the (bio)cathode, thus completing the circuit. Additionally, both electrodes are equipped with highly capacitive materials, such as conducting and carbon nanotubes, to enhance their capacitive properties and store charges. This wearable hybrid biodevice can function as a BFC, a supercapacitor, and a self-powered glucose biosensor, providing adequate energy conversion and self-charging through the enzymatic oxidation of glucose biofuel.

After analyzing the previous research, sweat is considered one of the best samples for monitoring parameters in POC. Unlike blood and interstitial fluid sampling, the principal reason for its usability is that sweat is collected noninvasively and avoids contamination, irritation, and issues arising from the use of other non-invasive biofluids such as saliva, tears, and urine. Also, wearables like microfluidic patches can perform real-time and cost-effective analysis in nearly any setting without requiring trained personnel. Moreover, beyond the literature reviewed up to now, the in situ analysis of sweat samples could be applied to a broad range of medical applications, including managing kidney disorders, tracking immune responses and stress levels, and guiding the use of prescription drugs [18].

Going one step further, Sun et al. (2021) [19] have developed a nanosystem that enables the diagnosis of scurvy from a single drop of raw (human) serum. This fully integrated point-of-care testing device is simple, handheld, pump-free, and affordable. It uses a bendable biofuel cell (BFC) and allows for the immediate analysis and accurate screening of scurvy with just 0.20 μL of raw serum. The system eliminates the need for sample pretreatment, facilitating scurvy diagnosis while accelerating analysis. Moreover, the system has a flexible, disposable, and pump-free vitamin C/air microfluidic BFC for self-powered vitamin C biosensing and a custom mini digital LED voltmeter for signal processing and transmission. Its design minimizes vitamin C degradation and ensures quick and reliable results.

Lastly, there is a growing interest in using standalone paper-based solutions [20,21], quantitative or qualitative readout solutions [22,23], and POC [24] devices due to their simplicity, low cost, portability, and availability. Some examples of such devices include Total Self-Powered Electrochromic Systems without electronics (Pellitero et al. (2018) [25]) based on the ohm-ic drop, where the color front relates directly to the analyte concentration. Likewise, Hickey et al. (2016) present another standalone lactate detection method that does not require a specific power source [26], acting as a sensor capable of generating a power density in the range of 125 µWcm^−2^. However, the main drawback is the sensor’s inability to extract the measurements for processing and translation to a database without using an external smartphone or other resources [27].

A self-powered paper-based device has been developed by Fraiwan et al. (2015) [28], which can monitor glucose levels using an enzymatic fuel cell (EFC). This device could be an exciting solution for resource-limited and remote regions [1]. The 3D paper-based EFC requires only a tiny sample (just a single drop of 20 μL) that can be pulled by the capillary force through patterned microfluidic pathways on paper. The sensor can be read using a simple digital multimeter. Instead of using an enzymatic cathode, an air-breathing cathode on paper is used, which provides a lower cost and higher practicality in developing countries. Also, Fraiwan et al. (2016) [29] developed a paper-based microbial fuel cell (MFC) that can be stacked and integrated to serve as an alternative power source. The 3D MFC stack was created by folding 2D paper sheets and sandwiching them between multifunctional layers to create a two-chamber configuration. The MFC stack generated a maximum power density of 1.27 μW/cm^2^ and an open circuit voltage of 1.6 V through microbial metabolism with just a 50 μL drop of anolyte and catholyte.

Similarly, Pal et al. (2017) [22] developed a self-powered, paper-based electrochemical device (SPED), enabling its use in low-resource settings for sensitive diagnostics. The device’s top layer comprises cellulose paper with patterned hydrophobic domains that separate hydrophilic, wicking-based microfluidic channels. This layer has self-pipetting test zones for electrochemical detection. The bottom layer is a triboelectric generator (TEG) made of hydrophobic paper, which can harvest electric energy from the user’s touch. The SPED interfaces with a rechargeable handheld potentiostat, which enables the accurate quantitative electrochemical detection of glucose, uric acid, and L-lactate. The battery powering the potentiostat can be recharged by the user using the sequential discharge of a capacitor previously charged with the TEG built into the SPED. A specially developed diagnostic application can automatically identify and quantify each colorimetric test from a digital SPED image. This procedure makes it easy to perform accurate and reliable diagnostic tests in low-resource settings.

Montes-Cebrián et al. (2018) [30] introduced the ‘Plug-and-Power’ concept, a novel paper-based approach that uses a serum sample with a minimum volume of 12.5 μL. They created a plug-and-play reader-disposable blood glucometer that utilizes a disposable test component to power a single test. The reader unit, which contains all necessary electronic systems to complete the test and display the result, is independent of any battery or power source. The disposable test component serves as both the sensor and power source and consists of a test strip containing a paper-based power source and a paper-based BFC as the glucose sensor. The battery-free electronic reader extracts energy from the test strip, samples and processes the signal provided, and displays the glucose concentration in the sample (see Figure 3).

Although all the examples mentioned thus far are still works of research, exploring some patents and commercial products in the field is fascinating in order to see the practical application of this knowledge. In 2018, Sang Jae et al. (2018) filed a patent [31] for a self-powered glucose sensor that detects the glucose concentration using a piezoelectric nanogenerator powering scheme. A glucose sample interacts with the glucose sensor, which has an active layer between the first and second electrode layers, making contact with the active layer. The active layer is a film containing BaTiO_3_, which generates an electrical signal when subjected to the piezoelectric force induced by applying a mechanical load. The value of the generated electrical signal changes depending on the concentration of glucose chemisorbed on the active layer.

Another patent example is the one filled by Wang et al. (2020) [32]; a patent for self-powered biosensors that can carry out the sensing of metabolites while using power from a biofuel. The patent includes self-powering BFCs and sensor devices, systems, and techniques to achieve metabolite sensing while utilizing biofuel energy to directly drive an analog-to-digital converter and wireless transmitter. The patent also covers ingestible devices that work on the same principle.

Rabost García et al. (2020) filed a patent [33] for a microfluidic system and method for continuously monitoring metabolites and (or) the properties of biofluids. The system uses passive capillary valves and pumps, enabling an autonomous and discontinuous measurement process for extended periods. It comprises at least one measuring chamber, one inlet for the input of a biofluid, a microfluidic intake channel fluidly connecting the inlet with the measuring chamber, and one sensor suitable for measuring a parameter of an analyte of a biofluid. A passive fluid pump is connected to the measuring chamber and generates capillary pressure higher than the biofluid generation pressure. A retention valve interposes between the measuring chamber and the fluid pump. It stops the biofluid flow for a specific period of time when the measuring chamber is filled with biofluid. Thus, this device collects and transports a biofluid, preferably sweat, for repetitive and electrochemical measurements.

Currently, Onalabs [34], a company specializing in continuous non-invasive monitoring, owns the patent. The company has two main focuses: lactate and glucose monitoring. For lactate monitoring, they have developed a lactate reader designed for athletes, which can continuously monitor their lactate levels through sweat. The device is a patch that can be easily applied to the pectoral area and connected to the athlete’s smart sports watch via Bluetooth. It can also connect to other devices with Bluetooth connectivity, such as a mobile phone. Similarly, the technology patented [35] by A. Álvarez-Carulla et al. (2021) [36] is a fuel cell-based POC system. In this approach, the system employs a single source energy generator and sensor element that effectively measures and extracts energy to power the electronics, using actual fuel cells and emulsifiers of ethanol, lactate, and methanol.

Finally, Figure 4 summarizes the temporal development of milestones and key enabling technologies for self-powered POC applications throughout the analyzed period.

## 4. Key Enabling Technologies

It is crucial to identify the key enabling technologies that drive advancements in self-powered POC devices after describing the most outstanding advances.

A self-powering scenario refers to a process in which the sensing process powers the sensing device. Electrochemical energy conversion is the most common method of self-powered sensing, which involves using an analyte’s chemical source reaction to produce electrical energy. The amount of analyte determines the total energy produced in the response [1]. Biofuel cells (BFCs) are one of the most prominent examples of this technology.

Conventional fuel cells convert chemical energy from fuel into electrical energy through electrochemical processes. They typically consist of fuel, oxidant, anodic and cathodic substrate materials, and a semipermeable membrane that separates the anode and cathode. However, BFCs differ from conventional fuel cells as they use natural proteins or microorganisms as biocatalysts for the anodic and cathodic substrate materials. These biocatalysts have high electrocatalytic activity at moderate pH and temperature levels, making them renewable and capable of oxidizing various fuels. In recent years, other types of biofuel cells have been developed and can be categorized as follows [37]:

- A primary fuel is used by a BFC, generating a material such as hydrogen, which, in turn, is a secondary fuel used by a conventional hydrogen/oxygen fuel cell.
- An organic fuel, such as glucose, is used in a BFC and directly generates bioelectricity. This BFC can contain enzymes or microorganisms.
- Photochemically active systems and biological moieties are used to harvest energy from sunlight and convert it to electrical power (energy).

**Figure 5:**
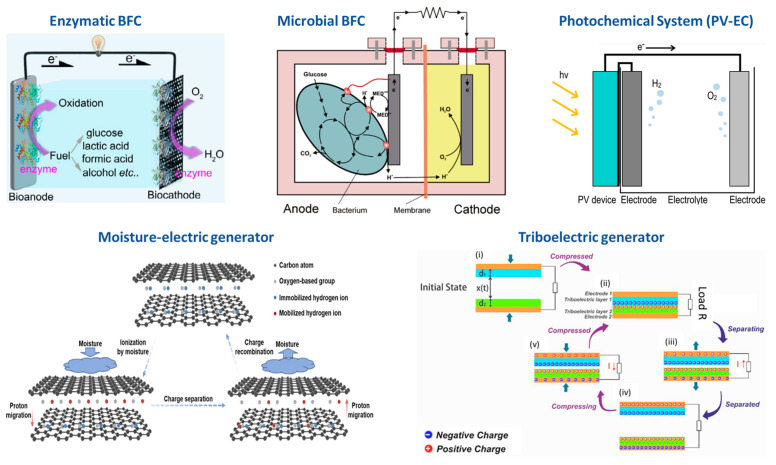
Overview of technologies for self-powering POC scenarios: the enzymatic BFC [38], microbial BFC [46], photochemical converter [47], moisture generator [42], and triboelectric effect generator [44].

**Figure 1:**
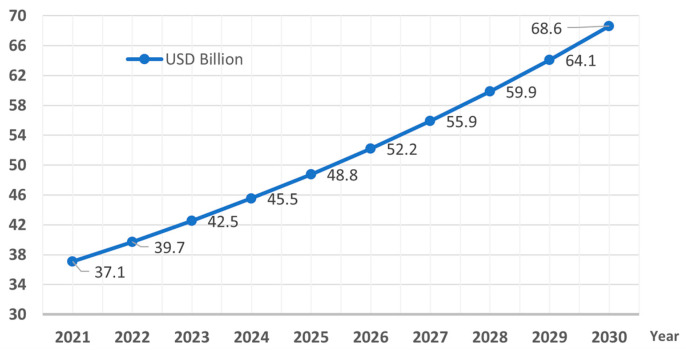
The market size of POC diagnostics, 2021 to 2030 (USD Billion).

**Figure 2:**
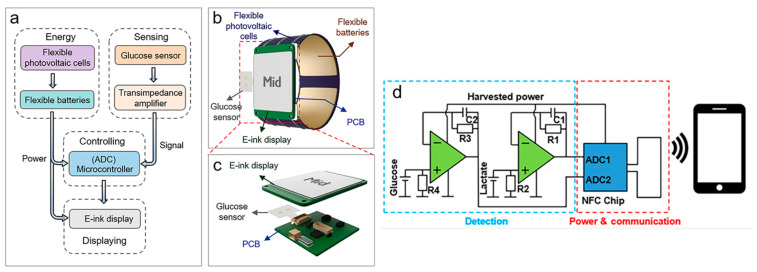
(**a**) System-level block diagram of the self-powered smartwatch using solar cell powering. (**b**,**c**) Illustrations of the self-powered smartwatch components. Adapted from [10]. (**d**) Schematic of the battery-free sweat analysis POC subsystems energized via a NFC link. Adapted from [11].

**Figure 3:**
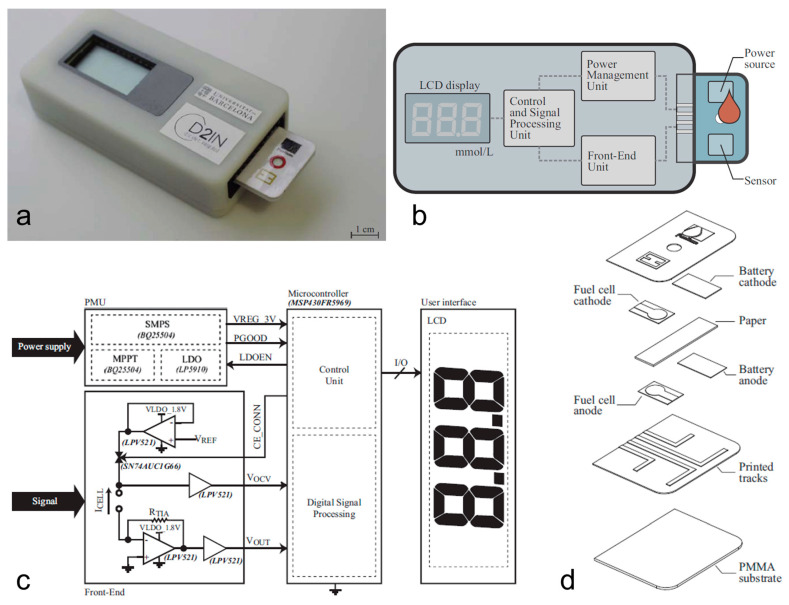
The ‘Plug-and-Power’ concept. (**a**) Battery-free electronic reader device with the disposable test component, (**b**) General architecture overview. (**c**) Electrical schematic details. (**d**) Disposable cartridge details of the paper-based power source and BFC glucose sensor. Adapted from [30].

**Figure 4:**
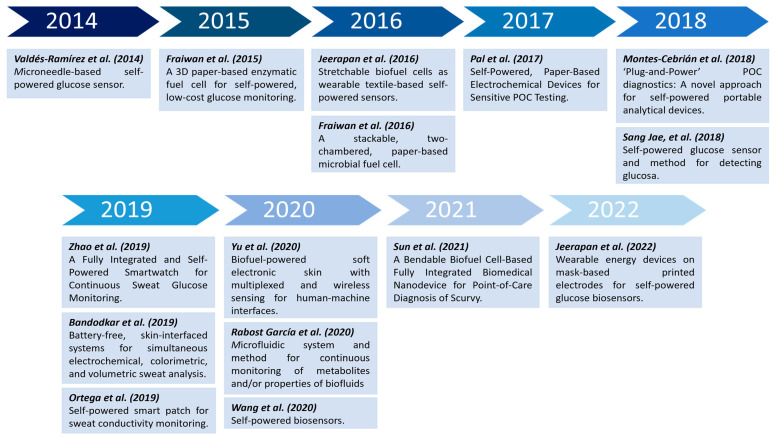
Evolution of the outstanding achievements of the reviewed state-of-the-art POC technology [9,10,11,12,13,14,17,19,23,28,29,30,31,32,33].

Enzymatic biofuel cells (EBFCs) are a specific type of BFC (Figure 5) that use biological enzymes as electrode catalysts [38]. These enzymes are exposed to the reaction system without cell membrane protection, which results in their degradation. Despite this, these biocatalysts are highly specific and can avoid cross-reactions. Additionally, they have high catalytic activity and pose no risks to biological bodies. Due to the advances in biocompatibility, high efficiency, and miniaturization, two cutting-edge applications have emerged: (1) implantable EBFCs, which obtain energy from the blood or similar body fluids to power implanted devices such as pacemakers, and (2) self-powered biosensors that can monitor and regulate health conditions depending only on changes in the EBFC’s power output [38].

In the last decade or so, the scientific community has been intrigued by microbial fuel cell (MFC) technology (Figure 5), which shows potential in converting organic waste into electricity through electrochemical reactions catalyzed by microorganisms at the anode and microorganisms, enzymes, or abiotic materials at the cathode [39]. In MFCs, bacteria oxidize a substrate in the anode, releasing electrons that are transferred to the cathode via a conductive material. In the cathode, the electrons react with oxygen, while the protons diffuse through a proton exchange membrane. For MFCs to work, electrons must be continuously released in the anode and consumed in the cathode. The bacteria’s metabolic energy gain is directly proportional to the difference between the anode and the substrate’s redox potential. The optimal design for an MFC is still under investigation. Currently, researchers are developing different electron materials and more selective membranes for proton exchange to enhance performance [40].

Alternatively, solar energy can be converted into chemical fuels through photochemical systems such as H_2_ and carbon-based fuels. H_2_ can be produced by splitting water, while carbon-based fuels can be synthesized by reducing CO_2_. Research on solar-to-fuel (STF) production has rapidly developed over the past few decades, leading to the emergence of several techniques such as photocatalytic, photovoltaic-electrochemical (PV-EC) (Figure 5), photo-electrochemical (PEC), and solar thermochemical systems. In these systems, solar energy is harnessed to directly drive chemical reactions or indirectly drive them after being converted into other forms of energy, such as electrical or thermal [41].

These systems (and derivations of BFCs) generate bioelectricity through renewable energy sources, making them safer and more efficient than Li-ion batteries and direct methanol fuel cells and able to produce a higher energy density. These features enable various applications, making them the next generation of energy devices. In their recent study, Álvarez-Carulla et al. (2021) [36] proposed a novel electronic architecture approach that utilizes a fuel cell to extract power and perform a sample concentration measurement simultaneously. The system employs a maximum power point tracking (MPPT) algorithm to extract power efficiently. The proposed approach addresses three critical issues related to self-powered POC devices that use a single fuel cell as both a sensor and a power source: (1) simultaneous power extraction and sample concentration measurement, (2) efficient power extraction, and (3) fuel cell biasing. Thus, the system solves three major issues in the current state-of-the-art. The entire architecture manages the powering, biasing, and measuring, and it also can control the processing and the output data interface for a POC. The device demonstrated its ability to prioritize energy extraction in terms of efficiency and extract concentration measurements for various scenarios. The efficiency, start-up time, transfer functions, and concentration detections of ethanol, lactate, and methanol-based fuel cells were thoroughly characterized. The minimum efficiency of ethanol-based fuel cells was 95%, while lactate-based fuel cells were 90% efficient, and methanol-based fuel cells were 80% efficient. The maximum start-up time was 10 s, 12 s, and 0.5 s, respectively. All fuel cells had a power consumption lower than 36 μW.

One step further, moisture-electric generators (MEGs) have recently gained attention as a viable source of clean energy due to their ability to harvest and convert moisture into electricity (Figure 5). They have a simple device setup and are eco-friendly, making them an attractive option for energy conversion. It is estimated that 50% of the absorbed solar energy on Earth is used for water evaporation, making MEGs an excellent opportunity to capture this renewable energy and convert it into electricity [42]. MEGs work by absorbing water molecules from moisture and releasing mobilized ions to generate separated charges for the generation of electricity. Currently, most MEGs use carbon-based materials such as carbon nanotubes, carbon nanoparticles, and graphene oxide (GO), which are abundant on Earth and good for the environment. GO shows the most promising potential of all the materials due to its high specific surface area, abundant oxygen-based groups, excellent moisture absorption, and good mechanical features. Although the MEG is still under research, an Australian company, Strategic Elements, has already developed a self-rechargeable battery, called Energy Ink^TM^, that generates electricity from moisture, either from the air or the skin surface [43]. 

Finally, in recent years, mechanical energy harvesters such as piezoelectric generators and triboelectric (TEGs) have become increasingly popular for high-efficiency energy harvesting. They are used to develop self-powered systems and as sensors [44]. These systems charge a material surface when it comes into proximity or contact with another material. When two different materials meet, they develop equal and opposite charges based on their surface properties. The opposite charges are formed on the back electrodes via electrostatic induction, which redistributes electrical charges due to the influence of nearby charges, resulting in a potential difference. In turn, it helps to produce an AC output by allowing the flow of electrons between the electrodes. TEGs can be treated as devices whose capacitance varies, and they produce electricity through physical contact, which can be solid–solid or solid–liquid contact. To date, triboelectric generators (TEGs) have four operating modes: vertical contact separation (C-S), lateral sliding (LS), single electrode (SE), and freestanding layer triboelectric (FT) generators (Figure 5). Although these modes work similarly, they differ in design, relative motion, applied force, and performance [45].

## 5. SWOT Analysis of the Technology

Next, a study of potentialities is carried out. For that, a SWOT analysis (short for strengths, weaknesses, opportunities, and threats) is a well-established strategy to compare technologies, features, and companies (among other items) with each other. It helps differentiate and establish a niche within the broader market. Table 1 summarizes the SWOT analysis for self-powered POC devices, identifying and analyzing the technology’s internal and external solid and weak points compared to the current market situation. 

## 6. Conclusions

To achieve power-free, fully portable POC devices suitable for all situations and places, it is necessary to develop reliable and robust self-powered biosensors. These self-powered POCs could replace the potentiostat and other high-power consuming electronics, simplifying the whole system and achieving a more user-friendly device, which would be optimal for bridging the technological gap across generations and regions. BFCs are an eco-friendly option currently being widely investigated and work as self-powered sensors, fulfilling the role of biosensors and power sources. As has been seen in the review of the state-of-the-art and the technologies that enable these POCs, there is an increase in promising research in this field, and several promising applications involving nanostructured screen-printed electrodes, paper-based batteries powered by sweat, or wearable devices self-powered by different BFCs, have been reported. Nevertheless, self-powered POCs have significant drawbacks to overcome, such as the instability of enzymes or other biocomponents and the reduced lifetime of the power supply because of the need to achieve the energy demands of most sensors and applications.

Many academic publications have appeared recently, and remarkable progress has been achieved. Nevertheless, very few new products are available, demonstrating the need to bridge the gap between academic research and the market to bring innovation to the industry. Many issues still need to be addressed in POC devices, particularly biosensor technology. Some relevant problems, such as sensitivity, specificity, and instability, are well-known and intrinsic to the biological elements used in the biosensor. Other issues to be solved are the automation achievable by increasing the lifetime of these self-powered systems, which still have room for improvement, ensuring the transmission of a high volume of data without interference in signal processing, and improving the speed of data communication. Furthermore, attention to the data so that the user of the POC device can manage to avoid being overwhelmed by unnecessary information is necessary, and there is an urgent need to develop practical security algorithms to protect data transmission and storage, for which blockchain technology has the chance to play a vital role in a near future.

Finally, the high costs related to R&D and the ethical and legal aspects involved in these processes, such as clinical trials and regulatory approvals, especially hard to obtain in the medical industry, are other significant barriers that slow down the realization of self-powered POCs in final market applications. But, despite this, new companies based on the power-free POC concept have already started to emerge, introducing products for which power generation comes directly from the sample and powers the necessary electronic systems without any other type of power source.

## Figures and Tables

**Table 1:** SWOT analysis of self-powered devices.

Strengths	Weaknesses
Low weight and reduced, compact, and portable sizes. Long-term operation. Elimination of battery dependencies. Affordable and adequate for developing countries. Reduction of contaminating electricity consumption (eco-friendly). Allows us to perform an early stage diagnosis of diseases. Suitable for use everywhere since there is no need for electricity sources. Simplification of the system’s electronics; components like potentiostats can be avoided.	Potential issues with the electronic circuit that turn off the whole product. Still under research, need for further validation and commercialization. Relatively new approaches to electricity sources can give rise to errors or distrust. Privacy leakage is especially sensitive as it deals with health information. Reduced lifetime of the power supply since it does not meet many applications’ energy demands. Enzymes’ instability (in EBFCs) or the instability of other biocomponents.
**Opportunities**	**Threats**
Capacity to monitor several body locations at the same time. A growing market with significant investments and expected to increase exponentially in the next few years. Connection to the Internet of Things provides real-time information to healthcare professionals. Few commercial products in the market. Bridging the technological gap. Can store data and generate medical records for future consultations with doctors. Can work in combination with smartphones or other mobile communications to display and store data. Can target population growth and aging, dealing efficiently with chronic conditions.	The reluctance of patients and healthcare professionals. Problems with wireless communication include a loss of data, data security, and relatively slow sampling rates. Lack of maintenance facilities.
